# Risk factors for hospitalized respiratory syncytial virus disease and its severe outcomes

**DOI:** 10.1111/irv.12729

**Published:** 2020-02-16

**Authors:** Wei Cai, Silke Buda, Ekkehard Schuler, Siddhivinayak Hirve, Wenqing Zhang, Walter Haas

**Affiliations:** ^1^ Respiratory Infections Unit Department for Infectious Disease Epidemiology Robert Koch Institute Berlin Germany; ^2^ Medizinische Fakultät Charité – Universitätsmedizin Berlin Germany; ^3^ HELIOS KLINIKEN GmbH Berlin Germany; ^4^ Global Influenza Programme World Health Organization Geneva Switzerland

**Keywords:** comorbidity, hospitalization, intensive care units, international classification of diseases, logistic models, respiratory syncytial virus, risk factors, ventilation

## Abstract

**Introduction:**

Respiratory syncytial virus (RSV) is a major cause of hospital admission for acute lower respiratory tract infection in young children.

**Objectives:**

We aimed to identify risk factors for hospitalized RSV disease and its severe outcomes.

**Methods:**

We conducted a retrospective cohort study analyzing data of a ICD‐10‐code‐based hospital surveillance for severe acute respiratory infections (SARI). Using univariable and multivariable logistic regression analysis, we assessed age‐group, gender, season, and underlying medical conditions as possible risk factors for RSV and its severe outcomes including ICU admission, application of ventilator support, and death, respectively.

**Results:**

Of the 413 552 patients hospitalized with SARI in the database, 8761 were diagnosed with RSV from week 01/2009 to 20/2018 with 97% (8521) aged <5 years. Among children aged <5 years, age‐groups 0‐5 months (OR: 20.29, 95% CI: 18.37‐22.41) and 6 months‐1 year (OR: 4.59, 95% CI: 4.16‐5.06), and underlying respiratory and cardiovascular disorders specific to the perinatal period (OR: 1.32, 95% CI: 1.11‐1.57) were risk factors for being diagnosed with RSV. Age‐group 0‐5 months (OR: 2.39, 95% CI: 1.45‐3.94), low birth weight (OR: 6.77, 95% CI: 1.28‐35.71), preterm newborn (OR: 6.71, 95% CI: 2.19‐20.61), underlying respiratory and cardiovascular disorders specific to the perinatal period (OR: 4.97, 95% CI: 3.36‐7.34), congenital malformation of the heart (OR: 3.65, 95% CI: 1.90‐7.02), congenital malformation of the great vessels (OR: 3.50, 95% CI: 1.10‐11.18), congenital defect originating in perinatal period (OR: 4.07, 95% CI: 1.71‐9.70), cardiovascular disease (OR: 5.19, 95% CI: 2.77‐9.72), neurological disorders (OR: 6.48, 95% CI: 3.76‐11.18), blood disease (OR: 3.67, 95% CI: 1.98‐6.79), and liver disease (OR: 14.99, 95% CI: 1.49‐150.82) contributed to ICU admission in RSV cases.

**Conclusions:**

Using ICD‐10‐based surveillance data allows to identify risk factors for hospitalized RSV disease and its severe outcomes, and quantify the risk in different age‐groups.

## 1 INTRODUCTION

Respiratory syncytial virus (RSV) is a worldwide distributed pathogen of acute respiratory infection (ARI) of all ages. In infants and young children, RSV is the most common cause of acute lower respiratory tract infection (ALRI) and a major cause of hospital admission for ALRI. Worldwide, about 33 million RSV‐associated ALRI episodes occurred in children younger than 5 years, and about 3 million were severe enough to necessitate hospital admission in 2015.^1^, ^2^ RSV has been found to be an important cause of viral infection requiring pediatric intensive care unit (ICU) admission and ventilator support, as well as a frequent course of death in young children.^3^, ^4^

Currently, there are no licensed vaccines against RSV; only passive immunization with palivizumab is available for young children at high risk.^5^ In 2015, the World Health Organization Product Development for Vaccines Advisory Committee highlighted the development of RSV vaccines. Several novel RSV vaccines and long‐acting monoclonal antibodies have shown promising results in clinical trials and are expected to enter the market in a short‐medium term.^6^, ^7^

Risk groups of severe RSV will benefit most from the RSV immunization once RSV vaccines become available. So far, some studies indicated that besides some socio‐demographic and environmental factors including being male, household crowding, and passive smoking, underlying medical conditions such as prematurity, congenital heart disease, and chronic pulmonary and cardiovascular diseases were associated with RSV disease.^8^, ^9^, ^10^ Furthermore, certain underlying medical conditions predispose young children to severe RSV disease^9^, ^11^ which is likely to result in ICU admission with ventilator support and a higher risk of death.^12^ However, to our knowledge, the risk of underlying medical conditions has not been assessed comprehensively with respect to risk for RSV and its severe outcomes, and studies have shown inconsistent results for some underlying medical conditions.^10^

The development of RSV vaccination strategies and the evaluation of the effects of RSV vaccination in the future rely on timely epidemiological data and long‐term observation of epidemiological situation of severe RSV and risk factors for severe RSV through national and other large‐scale RSV surveillance systems. The Robert Koch Institute (RKI) established an International Statistical Classification of Diseases, 10th revision (ICD‐10)‐code‐based surveillance system for severe ARI (ICOSARI) in cooperation with a private hospital network in Germany in 2015. Data of respiratory hospitalizations have been collected through the ICOSARI.^13^, ^14^

The aim of the present study was to identify risk factors, in particular underlying medical conditions as risk factors, for hospitalized RSV disease and its severe outcomes based on the ICD‐10‐code‐based surveillance data.

### 1.1 Methods

We performed a retrospective cohort study based on secondary data analysis of the ICOSARI database. The hospital network of ICOSARI includes 45 original sentinel hospitals, and additional 42 hospitals joined the hospital network after 2013. In 2015, 84 sentinel hospitals of ICOSARI located in 13 of the 16 federal states of Germany, covered 4.3% hospitals, and accounted for 5.9% of hospitalized patients in Germany. Since 2015, digital data of hospitalizations with any of respiratory ICD‐10 code (chapter X: J00‐J99)^15^ as primary or secondary discharge diagnosis have been collected prospectively and updated weekly. For retrospective analysis, ICD‐10 datasets of ICOSARI for the years 2009 to 2014 were collected. The collected data contain information on age, gender, admission and discharge date, primary and all secondary ICD‐10 code discharge diagnoses, length of hospital and ICU stay (in days), length of ventilation (in hours), and discharge mode. Further detailed description of the ICOSARI methodology was published elsewhere.^13^

The ICOSARI system was approved by the RKI and HELIOS Kliniken GmbH data protection authorities. As ICOSARI involved no interventions and the analysis was based on anonymized data only, no ethical clearance was required for this study.^13^

We used the following case definitions:

- Severe ARI (SARI) case: any patient hospitalized with any of the ARI ICD‐10 codes J09‐J22 (J09‐J11: influenza, J12‐J18: pneumonia, J20: acute bronchitis, J21: acute bronchiolitis, J22: unspecified acute lower respiratory infection) as primary or secondary discharge diagnosis^13^, ^15^; If a patient was readmitted to hospital, the patient would be counted again.
- RSV case: SARI case diagnosed with any of the RSV‐specific ICD‐10 codes (J12.1: RSV pneumonia, J20.5: acute bronchitis due to RSV, J21.0: acute bronchiolitis due to RSV) as primary or secondary discharge diagnosis.^15^
- ICU‐admitted RSV case: RSV case ever admitted to an ICU during the hospital stay.
- Ventilated RSV case: ICU‐admitted RSV case ever required ventilator support during the hospital stay.
- Deceased RSV case: RSV case died in hospital.

ICU admission, application of ventilator support, and death were considered markers of severe outcomes of hospitalized RSV disease.

Due to the possible inconsistent recording practices on ICU admission and application of ventilator support in the sentinel hospitals (personal communication), data on ICU admission and application of ventilator support from the original sentinel hospitals before 2013 and from the additional sentinel hospitals before 2015 were excluded from our data evaluation.

The underlying medical conditions evaluated in our study were neonatal disorders (disorder of newborn related to slow fetal growth and fetal malnutrition), extremely low birth weight (<1000 g), low birth weight (1000‐2499 g), extreme immaturity of newborn (<28 weeks), preterm newborn (28‐37 weeks), respiratory and cardiovascular disorder specific to the perinatal period (eg, intrauterine hypoxia, birth asphyxia, respiratory distress of newborn, congenital pneumonia, neonatal aspiration syndromes, interstitial emphysema, pulmonary hemorrhage), congenital disorders (congenital malformation of the heart, congenital malformation of the great vessels, congenital defect originating in perinatal period, Down syndrome, sickle‐cell disorder, cystic fibrosis), other comorbidities (vitamin D deficiency, asthma, chronic obstructive pulmonary disease (COPD), chronic pulmonary disease (excl. asthma and COPD), diabetes, cardiovascular disease, neurological disorders, blood disease (eg, nutritional anemias, coagulation defects, purpura, and other hemorrhagic conditions), renal failure, liver disease, tuberculosis, cancer, HIV/AIDS), and pregnancy. The underlying medical conditions could be primary or secondary discharge diagnoses. The specific ICD‐10 codes of the medical conditions chosen for our data evaluation are listed in Table 1, which were adapted from the Elixhauser and Fleming Comorbidity Indices.^16^, ^17^

**Table 1:** ICD‐10 codes of underlying medical conditions (adapted from Elixhauser and Fleming Comorbidity Indices)

Medical condition	ICD‐10 code
Disorder of newborn related to slow fetal growth and fetal malnutrition	P05.‐
Extremely low birth weight (<1000 g)	P07.0‐
Low birth weight (1000‐2499 g)	P07.1‐
Extreme immaturity of newborn (<28 weeks)	P07.2
Preterm newborn (28‐37 weeks)	P07.3
Respiratory and cardiovascular disorder specific to the perinatal period	P20‐P29
Congenital malformation of the heart	Q20‐Q24
Congenital malformation of the great vessels	Q25‐Q26
Congenital defect originating in perinatal period	Q02, Q30.‐, Q32‐Q37, Q44.‐, Q60.‐, Q61.‐, P70.0, P70.1, P70.2, P78.8
Down syndrome	Q90.‐
Sickle‐cell disorder	D57.‐
Cystic fibrosis	E84.‐
Vitamin D deficiency	E55.‐
Asthma	J45.‐, J46
Chronic obstructive pulmonary disease (COPD)	J44.‐
Chronic pulmonary disease (excl. asthma and COPD)	I27.8, I27.9, J40‐J43, J47, J60‐J67, J68.4, J70.1, J70.3
Diabetes	E10‐E14
Cardiovascular disease	A52.0, I05‐I08, I09.1, I09.8, I09.9, I10, I11, I13, I15, I25.5, I26, I27, I28.0, I28.8, I28.9, I34‐I39, I42.0, I42.5, I42.9, I43, I44.1‐I44.3, I45.6, I45.9, I47‐I50, P29.0, Q23.0‐Q23.3, R00.0, R00.1, R00.8, T82.1, Z45.0, Z95.0, Z95.2, Z95.4
Neurological disorders	G10‐G13, G20, G22, G25.4, G25.5, G31.2, G31.8, G31.9, G32, G35‐G37, G40, G41, G93.1, G93.4, R47.0, R56
Blood disease	D50.0, D50.8, D50.9, D51‐D53, D65‐D68, D69.1, D69.3‐D69.6
Renal failure	I12.0, I13.1, N18, N19, N25, Z49.0, Z49.2, Z94.0, Z99.2
Liver disease	B18, I85, I86, I98, K70, K71.1, K71.3, K71.5, K71.7, K72, K74, K76.0, K76.2, K76.9, Z94.4
Tuberculosis	A15‐A19
Cancer	C00‐C26, C30‐C34, C37‐C41, C43, C45‐C58, C60‐C85, C88, C96, C90.0, C90.2, C97
HIV/AIDS	B20‐B22, B24
Pregnancy	O00‐O99

For descriptive data analysis, we described the number of total RSV cases, ICU‐admitted RSV cases, ventilated RSV cases, and deceased RSV cases by age‐group (0‐5 months, 6 months‐1 year, 2‐4 years, 5‐64 years, ≥65 years), gender, calendar week, and underlying medical condition, respectively. The mean and median length of hospital stay, ICU stay and ventilation, and discharge mode were investigated, respectively.

We carried out univariable and multivariable logistic regression analyses to assess age‐group, gender, season, and underlying medical conditions as possible risk factors for being diagnosed with RSV among SARI cases, for ICU admission, application of ventilator support, and death among RSV cases, respectively. The univariable and multivariable logistic regression analyses were stratified in two age‐groups <5 years and ≥5 years. Underlying neonatal disorders were only evaluated as possible risk factors in the age‐group <5 years. Normally, relative risk (RR) should be calculated to measure the association between the exposure and the outcome in a cohort study. However, the odds ratios (OR) provides a reasonable approximation of the RR if the outcome is rare and occurs in less than 10% of the unexposed population.^18^ Our data met this condition that RSV cases were rare among SARI cases and RSV cases with different severe outcomes were rare among total RSV cases. Therefore, the ORs could be interpreted as RRs in our cohort study. ORs were calculated and presented with 95% confidence interval (95% CI). Two‐sided tests were applied. A *P* value of <.05 was considered statistically significant. Only variables indicating significant associations with RSV or any markers of severe outcomes of RSV in univariable logistic regression models were kept in multivariable logistic regression models, respectively.

We used Stata (version 15) and Microsoft Excel 2010 for the data analyses.

## 2 RESULTS

### 2.1 Study population (SARI cases)

**Figure 1:**
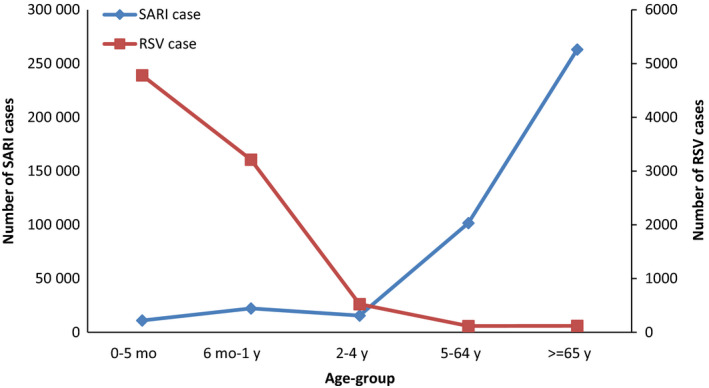
Number of SARI cases and RSV cases by age‐group, week 01/2009‐20/2018

**Figure 2:**
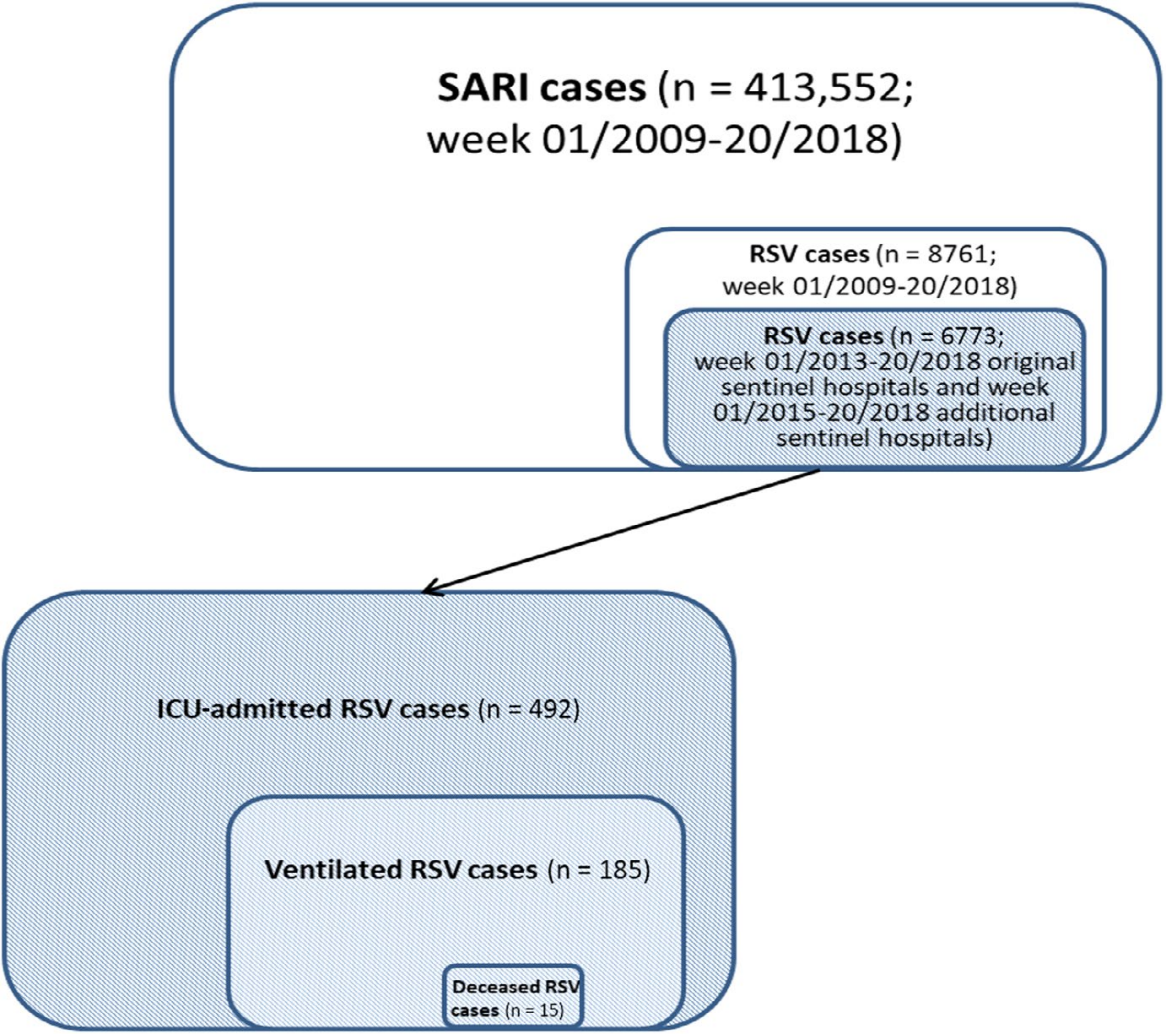
Number of SARI cases, RSV cases, ICU‐admitted RSV cases, ventilated RSV cases, and deceased RSV cases ever required ventilator support in ICU

A total of 413 552 SARI cases were identified from week 01/2009 to 20/2018. More than half (232 340, 56%) of them were male, and 64% (263 133) were ≥65 years old (Figures 1 and 2; Table 2). Of the SARI cases, 46% (188 948) received ARI (J09‐J22) as primary discharge diagnosis.

**Table 2:** Number of SARI and RSV cases and proportion of RSV cases in SARI cases by age‐group, week 01/2009‐20/2018

Age‐group	SARI case	RSV case
	n	n (%)
0‐5 months	11 014	4783 (43)
6 months‐1 year	22 207	3213 (14)
2‐4 years	15 530	525 (3)
5‐64 years	101 668	118 (0.1)
≥65 years	263 133	122 (0.05)
Total	413 552	8761 (2)

### 2.2 RSV cases

A total of 8761 (2%) RSV cases were identified. More than half (4955, 57%) of them were male. Most RSV cases were children aged <5 years (8521, 97%). The mean and median length of hospital stay were 6 and 5 days, respectively. Most (8599, 98%) RSV cases were discharged home, 2% (136) transferred to other facilities, and 0.3% (25) died in hospital. Of the RSV cases, 8228 (94%) received RSV‐specific ICD‐10 codes as primary discharge diagnosis (J12.1: 2995, 36%; J20.5: 2089, 25%; J21.0: 3144, 38%). Of the total RSV cases, 6773 were from the original sentinel hospitals after 2013 or from the additional sentinel hospitals after 2015 (Figures 1 and 2; Tables 2 and 3).

**Table 3:** RSV cases and RSV cases with severe outcomes by gender and age‐group (RSV cases and deceased RSV cases: week 01/2009‐20/2018; ICU‐admitted RSV cases and ventilated RSV cases: week 01/2013‐20/2018 original sentinel hospitals, week 01/2015‐20/2018 additional sentinel hospitals)

Age‐group	RSV cases		ICU‐admitted RSV cases		Ventilated RSV cases		Deceased RSV cases	
	Male	Female	Male	Female	Male	Female	Male	Female
	n (%)	n (%)	n (%)	n (%)	n (%)	n (%)	n (%)	n (%)
0‐5 months	2684 (54)	2099 (55)	164 (63)	164 (71)	61 (60)	53 (63)	0 (0)	3 (19)
6 months‐1 year	1875 (38)	1338 (35)	54 (21)	35 (15)	17 (17)	10 (12)	2 (22)	3 (19)
2‐4 years	273 (6)	252 (7)	12 (5)	9 (4)	3 (3)	5 (6)	1 (11)	1 (6)
5‐64 years	62 (1)	56 (1)	13 (5)	5 (2)	6 (6)	2 (2)	1 (11)	2 (13)
≥65 years	61 (1)	61 (2)	19 (7)	17 (7)	14 (14)	14 (17)	5 (56)	7 (44)
Total	4955	3806	262	230	101	84	9	16

### 2.3 ICU‐admitted RSV cases

Of the total RSV cases, 492 (7%) ICU‐admitted RSV cases were identified. More than half (262, 53%) of them were male, and 89% (438) were aged <5 years. The mean and median length of ICU stay were 9 and 5 days, respectively. During the ICU stay, 38% (185) required ventilator support. Most (449, 91%) ICU‐admitted RSV cases were discharged home, 6% (28) transferred to other facilities, and 3% (15) died in hospital (Figure 2; Table 3).

### 2.4 Ventilated RSV cases

Of the 185 ventilated RSV cases, more than half (101, 55%) were male, and 81% (149) were aged <5 years. The mean and median ventilation length were 211 and 112 hours, respectively. Most (152, 82%) ventilated RSV cases were discharged home, 10% (18) transferred to other facilities, and 8% (15) died in hospital (Figure 2; Table 3).

### 2.5 Deceased RSV cases

Of the 25 deceased RSV cases, more than half (16, 64%) were female, and nearly half (12, 48%) were ≥65 years old (Table 3). The mean and median length of hospital stay were 27 and 10 days, respectively.

### 2.6 RSV cases by calendar week

**Figure 3:**
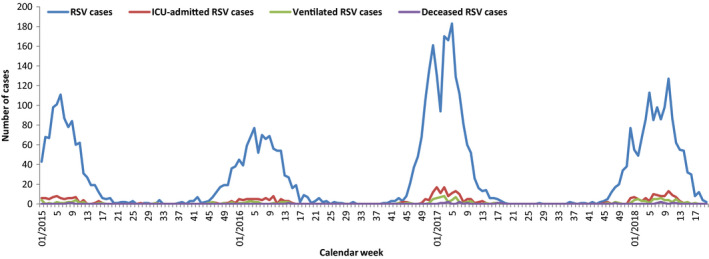
Number of RSV cases and RSV cases with severe outcomes by calendar week, week 01/2015‐20/2018

An overview of the number of total RSV cases and RSV cases with severe outcomes by calendar week is shown from week 01/2015 to 20/2018. During the study period, the most severe season was 2016/17 (Figure 3).

### 2.7 RSV cases with underlying medical conditions

The proportion of total RSV cases with different underlying medical conditions varied from 0% to 3.5%. Respiratory and cardiovascular disorder specific to the perinatal period (282, 3.5%) and cardiovascular disease (222, 2.5%) were the most common underlying medical conditions. They both were also the most common underlying medical conditions in the ICU‐admitted (60, 12.7%; 69, 14.0%) and ventilated RSV cases (35, 19.0%; 47, 25.4%). Cardiovascular disease (15, 60.0%) and blood disease (9, 36.0%) were the most common underlying medical conditions in deceased RSV cases. RSV cases with underlying sickle‐cell disorder (2), tuberculosis (2), and pregnant RSV cases (1; 28 years of age) were rare. No RSV case was diagnosed with HIV/AIDS (Table 4).

**Table 4:** RSV cases and RSV cases with severe outcomes by underlying medical condition (RSV cases and deceased RSV cases: week 01/2009‐20/2018; ICU‐admitted RSV cases and ventilated RSV cases: week 01/2013‐20/2018 original sentinel hospitals, week 01/2015‐20/2018 additional sentinel hospitals)

	N (%) of RSV cases n = 8761	N (%) of ICU‐admitted RSV cases n = 492	N (%) of ventilated RSV cases n = 185	N (%) of deceased RSV cases n = 25
*Medical condition*				
Disorder of newborn related to slow fetal growth and fetal malnutrition	6 (0.1)	1 (0.2)	0	0
Extremely low birth weight (<1000 g)	9 (0.1)	3 (0.6)	3 (1.6)	0
Low birth weight (1000‐2499 g)	51 (0.6)	16 (3.4)	12 (6.5)	0
Extreme immaturity of newborn (<28 weeks)	8 (0.1)	3 (0.6)	3 (1.6)	0
Preterm newborn (28‐37 weeks)	62 (0.8)	21 (4.4)	14 (7.6)	0
Respiratory and cardiovascular disorder specific to the perinatal period	282 (3.5)	60 (12.7)	35 (19.0)	1 (4.0)
Congenital malformation of the heart	131 (1.6)	35 (7.4)	19 (10.3)	2 (8.0)
Congenital malformation of the great vessels	34 (0.4)	13 (2.8)	8 (4.4)	0
Congenital defect originating in perinatal period	61 (0.7)	14 (2.9)	8 (4.3)	1 (4.0)
Down syndrome	42 (0.5)	9 (1.9)	2 (1.1)	0
Sickle‐cell disorder	2 (0.0)	0	0	0
Cystic fibrosis	9 (0.1)	1 (0.2)	1 (0.5)	0
Vitamin D deficiency	13 (0.2)	3 (0.6)	3 (1.6)	1 (4.0)
Asthma	63 (0.7)	4 (0.8)	1 (0.5)	0
Chronic obstructive pulmonary disease (COPD)	43 (0.5)	11 (2.2)	8 (4.3)	3 (12.0)
Chronic pulmonary disease (excl. asthma and COPD)	54 (0.6)	5 (1.0)	5 (2.7)	2 (8.0)
Diabetes	62 (0.7)	16 (3.3)	12 (6.5)	4 (16.0)
Cardiovascular disease	222 (2.5)	69 (14.0)	47 (25.4)	15 (60.0)
Neurological disorders	167 (1.9)	34 (6.9)	18 (9.7)	8 (32.0)
Blood disease	129 (1.5)	33 (6.7)	25 (13.5)	9 (36.0)
Renal failure	65 (0.7)	22 (4.5)	14 (7.6)	6 (24.0)
Liver disease	24 (0.3)	8 (1.6)	5 (2.7)	5 (20.0)
Tuberculosis	2 (0.0)	1 (0.2)	1 (0.5)	0
Cancer	27 (0.3)	4 (0.8)	2 (1.1)	1 (4.0)
HIV/AIDS	0	0	0	0
Pregnancy	1 (0.0)	0	0	0

### 2.8 Univariable analyses of risk factors for RSV and its severe outcomes

In the age‐group <5 years, the age‐groups 0‐5 months and 6 months‐1 year (reference group: 2‐4 years); being female; the seasons 2012/13, 2014/15, 2016/17, and 2017/18 (reference season: 2013/14); and underlying low birth weight, preterm newborn, and respiratory and cardiovascular disorder specific to the perinatal period were significantly associated with an increased risk of being diagnosed with RSV among SARI cases. Underlying congenital malformation of the heart and great vessels, congenital defect originating in perinatal period, cystic fibrosis, asthma, neurological disorders, blood disease, and cancer were significantly associated with a lower risk of being diagnosed with RSV. Age‐group 0‐5 months was significantly associated with ICU admission, whereas age‐group 2‐4 years was significantly associated with death among RSV cases. Gender and season were not associated with any severe outcomes of RSV. Most underlying medical conditions were significantly associated with severe outcomes of RSV.

In the age‐group ≥5 years, the age‐group 5‐64 years; the seasons 2012/13, 2014/15, 2016/17, and 2017/18; and underlying congenital defect originating in perinatal period, cystic fibrosis, vitamin D deficiency, asthma, and chronic pulmonary disease were significantly associated with an increased risk of being diagnosed with RSV among SARI cases. Underlying COPD, cardiovascular disease, and renal failure were significantly associated with a lower risk of being diagnosed with RSV. The age‐group ≥65 years was associated with death among RSV cases. Gender and season were not associated with any severe outcomes of RSV. Some medical conditions were significantly associated with severe outcomes of RSV.

### 2.9 Multivariable analyses of risk factors for RSV in age‐group <5 years

Among SARI cases aged <5 years, the age‐groups 0‐5 months (OR: 20.29, 95% CI: 18.37‐22.41) and 6 months‐1 year (OR: 4.59, 95% CI: 4.16‐5.06); being female (male: OR: 0.85, 95% CI: 0.80‐0.89); the seasons 2012/13 (OR: 1.26, 95% CI: 1.12‐1.41), 2014/15 (OR: 1.58, 95% CI: 1.42‐1.74), 2015/16 (OR: 1.14, 95% CI: 1.03‐1.26), 2016/17 (OR: 2.06, 95% CI: 1.88‐2.27), and 2017/18 (OR: 1.78, 95% CI: 1.61‐1.97); and underlying respiratory and cardiovascular disorder specific to the perinatal period (OR: 1.32, 95% CI: 1.11‐1.57) were significantly associated with an increased risk of being diagnosed with RSV. Underlying congenital malformation of the heart (OR: 0.69, 95% CI: 0.56‐0.85) and great vessels (OR: 0.38, 95% CI: 0.26‐0.58), congenital defect originating in perinatal period (OR: 0.41, 95% CI: 0.31‐0.55), asthma (OR: 0.40, 95% CI: 0.30‐0.53), neurological disorders (OR: 0.52, 95% CI: 0.43‐0.62), and blood disease (OR: 0.60, 95% CI: 0.48‐0.75) were significantly associated with a lower risk of being diagnosed with RSV (Table 5).

**Table 5:** Multivariable logistic regression analyses of risk factors for RSV and its severe outcomes in age‐group <5 years (RSV cases and deceased RSV cases: week 01/2009‐20/2018; ICU‐admitted RSV cases and ventilated RSV cases: week 01/2013‐20/2018 original sentinel hospitals, week 01/2015‐20/2018 additional sentinel hospitals)

	RSV case		ICU‐admitted RSV case		Ventilated RSV case		Deceased RSV case	
	OR	95% CI	OR	95% CI	OR	95% CI	OR	95% CI
Age‐group								
0‐5 months	**20.29**	**18.37‐22.41**	**2.39**	**1.45‐3.94**			0.28	0.03‐2.29
6 months‐1 year	**4.59**	**4.16‐5.06**	0.87	0.52‐1.48			0.63	0.10‐4.21
2‐4 years (reference group)	1		1				1	
Gender								
Male	**0.85**	**0.80‐0.89**						
Female (reference group)	1							
Season								
2009/10	**0.38**	**0.31‐0.45**						
2010/11	0.93	0.82‐1.07						
2011/12	0.90	0.79‐1.02						
2012/13	**1.26**	**1.12‐1.41**						
2013/14 (reference group)	1							
2014/15	**1.58**	**1.42‐1.74**						
2015/16	**1.14**	**1.03‐1.26**						
2016/17	**2.06**	**1.88‐2.27**						
2017/18	**1.78**	**1.61‐1.97**						
Medical condition								
Low birth weight (1000‐2499 g)	1.18	0.72‐1.93	6.77	1.28‐35.71	6.44	1.56‐26.55		
Preterm newborn (28‐37 weeks)	1.43	0.92‐2.24	**6.71**	**2.19‐20.61**	**3.91**	**1.20‐12.81**		
Respiratory and cardiovascular disorder specific to the perinatal period	**1.32**	**1.11‐1.57**	**4.97**	**3.36‐7.34**	**8.82**	**5.23‐14.89**		
Congenital malformation of the heart	**0.69**	**0.56‐0.85**	**3.65**	**1.90‐7.02**	**3.85**	**1.63‐9.13**	2.54	0.26‐24.78
Congenital malformation of the great vessels	**0.38**	**0.26‐0.58**	**3.50**	**1.10‐11.18**	1.87	0.46‐7.68		
Congenital defect originating in perinatal period	**0.41**	**0.31‐0.55**	**4.07**	**1.71‐9.70**	**3.30**	**1.05‐10.34**	2.61	0.23‐29.68
Down syndrome			2.61	0.93‐7.31				
Cystic fibrosis	0.26	0.06‐1.13			**35.13**	**1.76‐700.59**		
Vitamin D deficiency					9.02	0.88‐92.32		
Asthma	**0.40**	**0.30‐0.53**						
Chronic pulmonary disease (excl. asthma and COPD)					3.67	0.69‐19.46	**12.58**	**1.13‐140.15**
Cardiovascular disease			**5.19**	**2.77‐9.72**	**5.96**	**2.71‐13.08**	**9.42**	**1.46‐60.82**
Neurological disorders	**0.52**	**0.43‐0.62**	**6.48**	**3.76‐11.18**	**4.43**	**2.05‐9.56**	**21.70**	**4.98‐94.51**
Blood disease	**0.60**	**0.48‐0.75**	**3.67**	**1.98‐6.79**	**8.22**	**4.13‐16.36**	**12.17**	**2.23‐66.26**
Liver disease			**14.99**	**1.49‐150.82**	**13.70**	**1.81‐103.80**	**170.86**	**20.54‐1421.11**
Cancer	0.60	0.17‐2.07						

Note

Statistically significant results appear in bold.

#### 2.9.1 Multivariable analyses of risk factors for severe outcomes of RSV in age‐group <5 years

Among RSV cases aged <5 years, the age‐group 0‐5 months (OR: 2.39, 95% CI: 1.45‐3.94), underlying low birth weight (OR: 6.77. 95% CI: 1.28‐35.71), preterm newborn (OR: 6.71, 95% CI: 2.19‐20.61), respiratory and cardiovascular disorder specific to the perinatal period (OR: 4.97, 95% CI: 3.36‐7.34), congenital malformation of the heart (OR: 3.65, 95% CI: 1.90‐7.02) and great vessels (OR: 3.50, 95% CI: 1.10‐11.18), congenital defect origination in perinatal period (OR: 4.07, 95% CI: 1.71‐9.70), cardiovascular disease (OR: 5.19, 95% CI: 2.77‐9.72), neurological disorders (OR: 6.48, 95% CI: 3.76‐11.18), blood disease (OR: 3.67, 95% CI: 1.98‐6.79), and liver disease (OR: 14.99, 95% CI: 1.49‐150.82) were significantly associated with ICU admission (Table 5).

Underlying low birth weight (OR: 6.44, 95% CI: 1.56‐26.55), preterm newborn (OR: 3.91, 95% CI: 1.20‐12.81), respiratory and cardiovascular disorder specific to the perinatal period (OR: 8.82, 95% CI: 5.23‐14.89), congenital malformation of the heart (OR: 3.85, 95% CI: 1.63‐9.13), congenital defect originating in perinatal period (OR: 3.30, 95% CI: 1.05‐10.34), cystic fibrosis (OR: 35.13, 95% CI: 1.76‐700.59), cardiovascular disease (OR: 5.96, 95% CI: 2.71‐13.08), neurological disorders (OR: 4.43, 95% CI: 2.05‐9.56), blood disease (OR: 8.22, 95% CI: 4.13‐16.36), and liver disease (OR: 13.70, 95% CI: 1.81‐103.80) were significantly associated with application of ventilator support (Table 5).

Underlying chronic pulmonary disease (OR: 12.58, 95% CI: 1.13‐140.15), cardiovascular disease (OR: 9.42, 95% CI: 1.46‐60.82), neurological disorders (OR: 21.70, 95% CI: 4.98‐94.51), blood disease (OR: 12.17, 95% CI: 2.23‐66.26), and liver disease (OR: 170.86, 95% CI: 20.54‐1421.11) were significantly associated with an increased risk of death (Table 5).

### 2.10 Multivariable analyses of risk factors for RSV in age‐group ≥5 years

Among SARI cases aged ≥5 years, the age‐group 5‐64 years (≥65 years: OR: 0.53, 95% CI: 0.39‐0.71); the seasons 2012/13 (OR: 2.74, 95% CI: 1.38‐5.45), 2014/15 (OR: 2.15, 95% CI: 1.12‐4.15), 2016/17 (OR: 4.41, 95% CI: 2.43‐7.99), and 2017/18 (OR: 8.51, 95% CI: 4.78‐15.15); and underlying congenital defect originating in perinatal period (OR: 3.74, 95% CI: 1.19‐11.76), cystic fibrosis (OR: 13.40, 95% CI: 5.83‐30.78), vitamin D deficiency (OR: 2.57, 95% CI: 1.12‐5.93), and chronic pulmonary disease (OR: 2.04, 95% CI: 1.23‐3.38) were significantly associated with an increased risk of being diagnosed with RSV (Table 6).

**Table 6:** Multivariable logistic regression analyses of risk factors for RSV and its severe outcomes in age‐group ≥5 years (RSV cases and deceased RSV cases: week 01/2009‐20/2018; ICU‐admitted RSV cases and ventilated RSV cases: week 01/2013‐20/2018 original sentinel hospitals, week 01/2015‐20/2018 additional sentinel hospitals)

	RSV case		ICU‐admitted RSV case		Ventilated RSV case		Deceased RSV case	
	OR	95% CI	OR	95% CI	OR	95% CI	OR	95% CI
Age‐group								
5‐64 years (reference group)	1						1	
≥65 years	**0.53**	**0.39‐0.71**					**5.01**	**1.31‐19.15**
Season								
2009/10	0.60	0.17‐2.11						
2010/11	1.29	0.52‐3.21						
2011/12	1.65	0.73‐3.73						
2012/13	**2.74**	**1.38‐5.45**						
2013/14 (reference group)	1							
2014/15	**2.15**	**1.12‐4.15**						
2015/16	1.71	0.87‐3.39						
2016/17	**4.41**	**2.43‐7.99**						
2017/18	**8.51**	**4.78‐15.15**						
Medical condition								
Congenital defect originating in perinatal period	**3.74**	**1.19‐11.76**						
Cystic fibrosis	**13.40**	**5.83‐30.78**						
Vitamin D deficiency	**2.57**	**1.12‐5.93**						
Asthma	1.73	0.96‐3.12						
Chronic obstructive pulmonary disease (COPD)	0.75	0.50‐1.11						
Chronic pulmonary disease (excl. asthma and COPD)	**2.04**	**1.23‐3.38**						
Cardiovascular disease	0.71	0.53‐0.96						
Blood disease			**4.38**	**1.56‐12.27**	**3.40**	**1.22‐9.50**	**7.17**	**1.31‐19.15**
Renal failure	0.77	0.56‐1.06	**2.27**	**1.13‐4.55**				

Note

Statistically significant results appear in bold.

### 2.11 Multivariable analyses of risk factors for severe outcomes of RSV in age‐group ≥5 years

Among RSV cases aged ≥5 years, underlying blood disease was significantly associated with ICU admission (OR: 4.38, 95% CI: 1.56‐12.27), application of ventilator support (OR: 3.40, 95% CI: 1.22‐9.55), and death (OR: 7.17, 95% CI: 1.31‐19.15). Underlying renal failure (OR: 2.27, 95% CI: 1.13‐4.55) was significantly associated with ICU admission. The age‐group ≥65 years (OR: 5.01, 95% CI: 1.31‐19.15) was significantly associated with an increased risk of death (Table 6).

## 3 DISCUSSION

The ICOSARI surveillance data enabled us to describe epidemiology of hospitalized RSV in Germany and to identify risk factors for being diagnosed with RSV and severe outcomes of RSV in hospital. Besides the previously known risk factors for RSV and its severe outcomes including young age, certain underlying neonatal disorders, and chronic pulmonary and cardiovascular diseases, underlying cystic fibrosis and vitamin D deficiency were also found to be risk factors for being diagnosed with RSV. For severe outcomes of RSV, age‐group ≥65 years, underlying cystic fibrosis, neurological disorders, blood disease, liver disease, and renal failure were found to be risk factors.

In our study, the majority of SARI cases were older adults, whereas most RSV cases were young children. However, almost half of the deceased RSV cases were older adults. The ICU admission rate among hospitalized RSV cases (2%‐19%) and RSV fatality rate (0%‐5%) vary in different studies.^1^, ^12^, ^19^, ^20^, ^21^ Our results are within these ranges. In our study, the deceased RSV cases stayed in hospital in average longer than other RSV cases. RSV fatality rate was at least 10 times higher among ICU‐admitted and ventilated RSV cases than other RSV cases. The number of RSV cases was higher in the season 2016/17 during the study period which is in line with the finding of the RSV surveillance in the United States.^22^ The majority of RSV cases were without the underlying medical conditions in our study. However, in most deceased RSV cases, underlying cardiovascular disease was present.

The majority of RSV cases were aged <5 years in our study. Thus, we investigated risk factors separately in two age‐groups <5 and ≥5 years. To avoid confounding effects, we included in the multivariable models variables with significant association with RSV or its severe outcomes in the univariable models.

In the multivariable models for the age‐group <5 years, among SARI cases, children in the first months of life were significantly more likely to be diagnosed with RSV, and among RSV cases, they were more likely to be admitted to ICU. Our findings are concordant with the majority of reports that young age is a risk factor for hospitalization due to RSV^1^, ^2^, ^10^ and age below 3 months contributes to the increased severity of RSV disease.^23^ Being male is normally known to be a risk factor for RSV.^10^, ^24^ However, Grimwood et al reported non‐significant association between being male and RSV.^25^ In our study, being female was a risk factor for being diagnosed with RSV, and gender did not play a role in developing severe outcomes of RSV. The association between RSV and season varied across the 9 seasons. RSV was more likely to be diagnosed in the seasons 2016/17 and 2017/18.

Low birth weight and prematurity are widely recognized as important risk factors for RSV.^8^, ^9^, ^10^ In our study, they were not associated with being diagnosed with RSV, but were risk factors for ICU admission and application of ventilator support in RSV cases. Immature immune system, poorly developed airway, and reduced respiratory muscle capacity of premature infants may contribute to this risk.^9^, ^26^ Children with underlying congenital heart and great vessel disease, congenital defect originating in perinatal period, neurological disorders, and blood disease were significantly more likely to be diagnosed with other SARI compared to RSV in our study. However, the above‐mentioned underlying medical conditions, underlying cystic fibrosis, chronic pulmonary disease, cardiovascular disease, and liver disease still contributed to the severe outcomes among RSV cases. Our findings are in agreement with the reports that among infants, underlying congenital heart disease or lung disease increases severity of hospitalized RSV,^27^, ^28^, ^29^ and RSV infection is more severe in those with underlying cystic fibrosis.^9^, ^30^, ^31^ Poor growth and malnutrition are symptoms of cystic fibrosis which may affect pulmonary function and have an impact on the severity of RSV disease in infants with RSV and cystic fibrosis.^9^, ^31^

In the multivariable models for the age‐group ≥5 years, older adults were significantly unlikely to be diagnosed with RSV. However, they were significantly more likely to have severe outcome of death if they were diagnosed with RSV. This finding can be explained that RSV similar to seasonal influenza can cause severe respiratory complications in older adults, resulting in respiratory failure and high mortality.^32^ Gender was not associated with RSV or its severe outcomes. Like the age‐group <5 years, RSV was more likely to be diagnosed in the seasons 2016/17 and 2017/18. Although underlying cystic fibrosis normally increases severity of RSV in infants,^9^, ^30^, ^31^ in our study, it was also a risk factor for being diagnosed with RSV in older children and adults. Our results regarding vitamin D deficiency are in line with the findings that vitamin D may protect against RSV‐associated ALRI since vitamin D may influence the development of immune system, modulate early lung development, and decrease viral load during infection,^33^ but vitamin D deficiency is not associated with the increased severity of RSV.^34^

Underlying Down syndrome was reported as a risk factor for RSV in some studies,^10^, ^35^ whereas some other studies did not find the association.^10^, ^20^ In our study, it was like underlying disorder of newborn related to slow fetal growth and fetal malnutrition, diabetes, and cancer not associated with RSV or its severe outcomes in both age‐groups. The young age is associated with hospitalized RSV.^1^, ^2^, ^10^ However, children with Down syndrome that are admitted to the hospital tend to be older than children with RSV infection.^35^ This may partly explain our finding regarding the Down syndrome. RSV and other respiratory pathogens in early life play an important role in the inception and exacerbation of asthma.^36^, ^37^ We investigated underlying asthma as a possible risk factor for RSV and its severe outcomes. In age‐group <5 years, asthma patients were more likely to be diagnosed with other SARI compared to RSV, and in age‐group ≥5 years, no associations were found. However, RSV may be underdiagnosed in asthma patients due to similar clinical presentation, if no fever is present. COPD patients may be more susceptible to RSV infection^38^; however, we found no associations. In our study, the number of RSV cases with underlying sickle‐cell disorder, tuberculosis, and pregnant RSV cases was too low to support the data analyses. Underlying neurological disorders, blood disease, liver disease, and renal failure have been rarely investigated as possible risk factors for RSV or its severe outcomes in the literature. In our study, they contributed to the severe outcomes in RSV cases.

Our study has some limitations. Based on the hospitalization data alone, information on socio‐demographic factors except age and gender as well as environmental factors for RSV could not be captured. Thus, socio‐demographic and environmental factors and underlying medical conditions could not be evaluated in one model, which limited a comprehensive understanding of the risk factors for RSV and its severe outcomes. Further, no data were available to identify any children who had received palivizumab which would reduce the strength of association with risk factors. ICOSARI is a ICD‐10‐based syndromic surveillance system. Virological data of the ICOSARI network were not available. As the coding behavior of physicians may vary based on use of laboratory diagnostics and level of coding awareness, some true cases may be missed or wrongly included due to miscoding which could lead to information bias. RSV is also a common pathogen of ARI in older adults. In our study, among those aged ≥65 years, the proportion of RSV cases in SARI cases was lower than that reported in the literature.^39^ Our data suggest that in elderly SARI patients, RSV testing might be less frequently carried out. Thus, in our analysis we always use the expression being diagnosed with RSV not RSV infection. Also for this reason, we realized that we cannot exclude underdiagnosis of RSV, leading to underestimation of risks. Although the sample size of our study population and RSV cases was large, the number of deceased RSV cases and RSV cases with some underlying medical conditions was small which may lead to sparse data bias, especially for the evaluation of risk factors for deceased RSV cases and underlying liver disease as risk factor in age‐group <5 years. However, few other studies were large enough to look into the risk of death, whereas our approach using surveillance data, despite this limitation, allowed analysis of deceased RSV cases.

## 4 CONCLUSIONS

Using ICD‐10‐based surveillance data allows to identify risk factors for being diagnosed with RSV and severe outcomes of RSV in hospital, to quantify the risk in different age‐groups, and to monitor the risk routinely. Our findings will contribute to the development of a baseline for evaluating RSV vaccination strategies and RSV vaccination impact in the future, in particular on the target groups, and help reducing burden of RSV disease and its severe outcomes.

Further studies regarding risk factors for RSV are needed with the focus on the underlying medical conditions with inconsistent findings compared with the literature with consideration of socio‐demographic and environmental factors.

## CONFLICT OF INTERESTS

None.

## AUTHOR CONTRIBUTIONS

Wei Cai, Silke Buda, Walter Haas, Siddhivinayak Hirve, and Wenqing Zhang were involved in designing the study. Silke Buda and Ekkehard Schuler participated in the collection of ICOSARI data. Wei Cai analyzed the data and drafted the manuscript. All authors reviewed and approved the final manuscript.
